# Effects of Acute Methamphetamine on Emotional Memory Formation in Humans: Encoding vs Consolidation

**DOI:** 10.1371/journal.pone.0117062

**Published:** 2015-02-13

**Authors:** Michael E. Ballard, Jessica Weafer, David A. Gallo, Harriet de Wit

**Affiliations:** 1 Department of Psychiatry and Behavioral Neuroscience, University of Chicago, 5841 S. Maryland Ave., MC3077, Chicago, Illinois, 60637, United States of America; 2 Department of Psychology, University of Chicago, 5848 S. University Ave., Chicago, Illinois, 60637, United States of America; Rutgers University, UNITED STATES

## Abstract

Understanding how stimulant drugs affect memory is important for understanding their addictive potential. Here we examined the effects of acute d-methamphetamine (METH), administered either before (encoding phase) or immediately after (consolidation phase) study on memory for emotional and neutral images in healthy humans. Young adult volunteers (*N* = 60) were randomly assigned to either an encoding group (*N = 29*) or a consolidation group (*N* = 31). Across three experimental sessions, they received placebo and two doses of METH (10, 20 mg) either 45 min before (encoding) or immediately after (consolidation) viewing pictures of emotionally positive, neutral, and negative scenes. Memory for the pictures was tested two days later, under drug-free conditions. Half of the sample reported sleep disturbances following the high dose of METH, which affected their memory performance. Therefore, participants were classified as poor sleepers (less than 6 hours; n = 29) or adequate sleepers (6 or more hours; n = 31) prior to analyses. For adequate sleepers, METH (20 mg) administered before encoding significantly improved memory accuracy relative to placebo, especially for emotional (positive and negative), compared to neutral, stimuli. For poor sleepers in the encoding group, METH impaired memory. METH did not affect memory in the consolidation group regardless of sleep quality. These results extend previous findings showing that METH can enhance memory for salient emotional stimuli but only if it is present at the time of study, where it can affect both encoding and consolidation. METH does not appear to facilitate consolidation if administered after encoding. The study also demonstrates the important role of sleep in memory studies.

## Introduction

Drugs of abuse have direct effects on learning and memory, and these effects are thought to contribute to their abuse potential. Stimulant drugs in particular facilitate memory formation by acting on neural systems that guide learning about salient environmental stimuli [1,2,3]. Drug-induced activation of these systems results in strong associations between drug cues and drug reward, rendering drug-related stimuli highly significant and salient for the user. These associations in memory can persist long after withdrawal and after extended periods of abstinence, thus posing a risk factor for relapse years after initial sobriety. As such, a more comprehensive understanding of the precise ways in which stimulant drugs affect memory is critical to understanding their addictive properties.

Two distinct phases of memory formation that are potentially sensitive to stimulant drug effects are encoding and consolidation of to-be-remembered information. Encoding processes may include enhanced attention or initial processing of stimuli, whereas consolidation is a process of trace alteration and stabilization that occurs for a period of time after the initial exposure. In laboratory animals, stimulant drugs administered prior to stimulus presentation improve long-term memory (for a review, see [3], which may indicate actions during either encoding or consolidation, or both). However, stimulant drugs can also improve memory in rodents when they are administered immediately after stimulus presentation [4,5,6], indicating that they can affect memory consolidation, independent of any effects during encoding. In humans, stimulant drugs are known to facilitate memory when given before verbal learning (e.g., word lists) [7,8,9]. However, whether they exert this effect by affecting encoding or consolidation is not known. One previous human study has specifically examined stimulant effects on consolidation. [10] administered either 10 mg amphetamine (intramuscular) or placebo to healthy adults immediately following presentation of a list of unrelated neutral words, and tested memory for the word list one day later. Consistent with laboratory animal studies, amphetamine significantly increased the number of words recalled relative to placebo. However, participants performed an initial recall test 20 min after drug administration, and it is possible that enhanced encoding of the words during this initial recall test contributed to the drug’s reported effect on consolidation.

Few studies with humans have examined the effects of stimulant drugs on emotional memory, that is, memory for stimuli with positive or negative motivational value. This is a major gap in the literature, as several current theories of addiction postulate that addiction is related to maladaptive effects of drugs on emotional memory systems [11,12]. Additionally, memories with greater emotional valence are formed more readily and last longer than memories for neutral stimuli [13], and stimulant drugs are known to further heighten the perceived emotional valence of a stimulus [7,14]. Based on these observations, we hypothesized that a stimulant drug would preferentially enhance memory for emotional material, and that it would exert this effect during both the encoding and consolidation phases of memory formation.

In the present study, we examined the effects of the prototypic stimulant methamphetamine (METH) on emotional memory, separately during encoding and consolidation. One group of participants (encoding group) received placebo or METH (10, 20 mg oral) before viewing pictures with or without emotional content, and their memory for the pictures was tested two days later, under drug-free conditions. The other group (consolidation group) followed the same procedure, but they received placebo or METH immediately after picture viewing. Thus, in the encoding group we examined the drug’s effects on memory for information encountered while under the influence, which could include both initial encoding and post-encoding consolidation processes. In the consolidation group we examined the effects of METH on memory during the post-encoding consolidation process. We hypothesized that METH would enhance both encoding and consolidation, and that drug-induced memory facilitation would be more pronounced for emotional relative to neutral images.

Given that we tested memory two days after study sessions, it was important to consider participants’ sleep quality following drug administration. Methamphetamine users have impaired sleep quality and daytime sleepiness [15]. Sleep quality could be a potential confounding factor in analyses of drug effects on memory, as restful sleep is known to be important for late-stage memory consolidation [16]. This was especially important to consider, as there was the possibility that METH could disrupt sleep the night after dosing due to its relatively long half-life and prior evidence of sleep disruption following METH [17,18,19]. This could counteract drug effects on memory formation, resulting in no drug-induced enhancement, or even impairment following the drug. Because of this, sleep quality following each session was measured by self-report, and sleep quality was examined as a predictor of METH effects on memory.

## Methods

### Ethics statement

This study was approved by the Institutional Review Board of the University of Chicago and was carried out in accordance with the Declaration of Helsinki. All participants provided written informed consent for participation.

### Design

A mixed within- and between subjects design was used to test the effects of acute oral METH on encoding and consolidation of emotional memory. Healthy young men and women were randomly assigned to either the encoding group (*N* = 29; 15 female) or the consolidation group (*N* = 31; 16 female). All participants completed three 2-part experimental sessions, in which they received 0, 10, and 20 mg of METH in counterbalanced order under double-blind conditions. Each of the three sessions consisted of two laboratory visits—a five-and-a-half-hour encoding visit, followed two days later by a one-hour retrieval visit. During the encoding visits, participants ingested syrup containing drug or placebo and viewed a unique set of standardized pictures. The encoding group received placebo or METH before viewing images and received only placebo after picture viewing. The consolidation group always received placebo before picture viewing, and either placebo or METH immediately after. Memory was assessed two days later at the retrieval phase visit, during which no drugs were administered. Aside from drugs administered, the groups followed the same procedures, and research assistants were blind to group as well as specific drugs administered. Encoding phase visits began between 11am and 1pm so that the latest drug administration was at 2:30 pm. METH has a 4–6 hour biological half-life (Desoxyn [package insert] Deerfield, IL: Lundbeck Inc; 2009). The retrieval phase visits were conducted in the afternoons, between 12pm and 5pm.

### Subjects

Healthy volunteers aged 18–35 years were recruited from the University of Chicago and the surrounding community via posters, advertisements, and word-of-mouth referrals. Potential participants underwent a physician-supervised physical examination, including an electrocardiogram. They also completed a health questionnaire with detailed information on current and lifetime drug use, and were interviewed by trained clinical psychologists using a semi-structured psychiatric interview. Exclusion criteria included current Axis I DSM-IV disorder including any Substance Dependence other than Tobacco Dependence. Volunteers were excluded if they had a history of psychosis or mania, less than a high school education, lack of fluency in English, a body mass index outside of 19–26 kg/m^2^, high blood pressure (>140/90), an abnormal electrocardiogram, reported daily use of any medication other than birth control, or were pregnant, lactating, or planning to become pregnant in the next three months. Women not taking hormonal contraceptives were tested during their follicular phase only because hormonal fluctuations of the menstrual cycle can influence responses to the related drug dextroamphetamine [20].

### Procedure

Qualifying participants attended a one-hour orientation session to acquaint them with the study procedures and risks, provide informed consent, and practice study tasks and questionnaires. They were informed that the study was designed to examine the effects of drugs on mood and memory, and they were told that they might receive a placebo, stimulant (e.g., amphetamine), sedative/tranquilizer (e.g., Valium), or a marijuana-like drug. Participants were instructed to consume their normal amounts of caffeine and nicotine before sessions, but to abstain from using alcohol and prescription (other than birth control), over the counter, and illicit drugs for 24 hours before the sessions. They were informed that they would be tested for drug use before each session to verify abstinence. Participants were also advised to get their normal amounts of sleep, and not to eat solid food for two hours before the encoding phase visits to allow for proper drug absorption.

Participants were tested individually in comfortably furnished rooms with a television and VCR, magazines, and a computer for administering questionnaires and tasks. They were allowed to watch television, selected neutral movies, or read when no measures were being obtained, but they were not allowed to sleep, work, study, or use cell phones or internet. Upon arrival for each visit, participants first completed compliance measures including breath alcohol level (Alco-sensor III, Intoximeters, St. Louis, MO), and urine drug (ToxCup, Branan Medical Co. Irvine, CA) and pregnancy (women only, using an hCG assay; Aimstrip, Craig Medical, Vista, CA) tests.

A detailed timeline of the procedures taking place over a single experimental session is shown in Table 1. In brief, during the encoding phase visits, participants first completed compliance and baseline cardiovascular and mood measures, and then consumed 10 ml syrup with 100 mL water. For the encoding group, this syrup contained METH (10 or 20 mg) or placebo, and for the consolidation group it contained no drug. Forty-five minutes later (at the time of peak drug effects for encoding group participants [21]), participants viewed the pictures for which their memory would be tested two days later (see below for picture study procedures). Immediately after the last picture, participants consumed a second dose of syrup. For the encoding group this was always placebo, but for the consolidation group it contained placebo or METH (10 or 20 mg). Measures of subjective drug response, mood state, and cardiovascular function were obtained multiple times throughout the encoding phase visits (see ***Dependent measures***, below for details). Participants were allowed to leave when residual subjective and physiological drug effects had subsided. During the 1-hour retrieval phase visits, participants first provided compliance measures (including breath alcohol level (Alco-sensor III, Intoximeters, St. Louis, MO), and urine drug tests (ToxCup, Branan Medical Co. Irvine, CA), reported the number of hours they slept following the encoding phase visit, and completed baseline questionnaires. Then, they completed the recognition memory task. All participants were fully debriefed after study completion.

**Table 1:** Timeline of a single experimental session for encoding and consolidation groups.

**Encoding Phase**		
Group		Measure
Encoding	Consolidation	
Baseline	Baseline	BP/HR, POMS
0 min	-80 min	**TX 1: placebo (or METH [encoding group])**
+20 min	-60 min	BP/HR, DEQ, POMS
+40 min	-40 min	BP/HR, DEQ, POMS
+45 min	-35 min	*Picture encoding*
+80 min	-5 min	BP/HR, DEQ, POMS
+85 min	0 min	**TX 2: placebo (or METH [consolidation group])**
+100 min	+20 min	BP/HR, DEQ, POMS
+140 min	+60 min	BP/HR, DEQ, POMS
+200 min	+120 min	BP/HR, DEQ, POMS
+260 min	+180 min	BP/HR, DEQ, POMS
+320 min	+240 min	BP/HR, DEQ, POMS

Retrieval Phase		
BP/HR, POMS, *Picture retrieval*		

BP/HR: blood pressure & heart rate; POMS: profile of mood states; DEQ: drug effects questionnaire

### Dependent measures

**Memory Task: Encoding Phase**. This study employed the same picture memory procedure as previously described [22]. During each encoding phase visit, participants viewed a unique set of 60 pictures taken from the International Affective Picture System [23]. Each set was comprised of photographs depicting positive or pleasant, neutral, and negative or unpleasant scenes (20 each category), according to normative ratings. Pictures were displayed individually on a computer screen for 3000 msec, and were pseudorandomized such that no more than two pictures from the same valence category were presented consecutively. Participants rated each picture for perceived valence and arousal. Valence was defined as: 1) how positive, and 2) how negative the picture made them feel, and arousal was defined as how stimulated, excited, or awake they felt in response to the picture [24]. Valence was measured using the evaluative space grid [25]—a two-dimensional grid allowing independent ratings of positivity and negativity from zero (not at all) to four (extreme), and arousal was measured using Likert scales from one to nine.

**Memory Task: Retrieval Phase**. Two days after each encoding phase visit, participants returned to the laboratory to test their memory for the pictures viewed during the previous encoding phase visit. Participants were instructed to identify the pictures presented on the previous visit, from a set containing an equal number of novel (unstudied) distractor pictures, and to rate their confidence in their memory assessment using a Likert scale from 1 (not at all confident) to 9 (extremely confident). Current emotional response to each image was also rated. Raw outcome measures included hit rate (the proportion of studied pictures correctly recognized as studied at test), false alarm rate (the proportion of unstudied pictures incorrectly recognized as studied at test), and valence, arousal, and confidence ratings at test. The main outcome measure was memory accuracy, calculated by subtracting false alarm rate from hit rate. This method of subtracting false alarms from hits is widely used in order to control for changes in base rate responding [26].

**Subjective Measures: Drug Effects Questionnaire (DEQ)**. The DEQ consists of five questions concerning subjective drug response—specifically, how much participants currently feel a drug effect, like the drug’s current effects, dislike the drug’s current effects, feel high, and would be interested to take the same dose of the same drug again in the future [27]. Participants rated their responses on 100 mm sliding scales from zero “not at all / neutral” to 10 “very much.”

**Subjective Measures: Profile of Mood States (POMS)**. The POMS is a 72-item adjective checklist on which individuals report their current mood on a five-point scale from zero (not at all) to four (extremely) [28]. Eight clusters (scales) of items are separated empirically by factor analysis (Friendliness, Anxiety, Elation, Anger, Fatigue, Depression, Confusion, Vigor). Two summary scales are derived from the other scales: Arousal = (Anxiety + Vigor) − (Fatigue + Confusion); Positive Mood = Elation − Depression. Questions pertaining to ‘Friendliness’ and ‘Anger’ scales were not administered in order to reduce participant burden, and because these scales were not of primary interest in the present study.

**Physiological Measures**. Blood pressure and heart rate were measured using a portable digital blood pressure monitor (AND Medical/Life Source, San Jose, CA).

### Drugs

Dextromethamphetamine hydrochloride (Desoxyn; Lundbeck Inc., Deerfield, Illinois) 5 mg tablets were crushed and suspended in a flavored syrup at a concentration of 2 mg/mL and administered orally using 10 mL oral syringes (Baxa ExactaMed; Baxter Healthcare Corp., Deerfield, Illinois). The syrup, which is designed for administering drugs to children, consisted of equal parts Ora-Plus and Ora-Sweet (Paddock Laboratories Inc. Minneapolis, Minnesota). The same syrup without drug served as a placebo, and this was also used to dilute the METH stock syrup to a concentration of 1 mg/mL for the 10 mg dose condition. Accordingly, participants always received a single syringe containing 10 mL of syrup regardless of the dose being administered (0, 10, 20 mg METH). These are moderate doses of METH that are within the range prescribed for attention-deficit/hyperactivity disorder [29], and produce behavioral and subjective effects in healthy humans [21,30]. They are also within the range used nonmedically to increase wakefulness, enhance physical or cognitive performance, or for their euphorigenic effects. As noted above, METH and AMP are similar on physiologic and behavioral measures [31,32]. METH was administered in an oral suspension to achieve a rapid onset [21], to minimize the delay after encoding in the consolidation group [33].

### Statistical Analyses

**Memory task**. Memory task data were analyzed separately by group (encoding and consolidation) to determine METH’s effects on emotional evaluation and memory accuracy using two-way repeated-measures ANOVA with drug dose (0, 10, 20 mg) and picture valence (positive, neutral, negative) as the within-subjects factors. About half of subjects reported that the high dose of METH impaired their sleep following the memory task (see Results), and sleep is known to affect memory consolidation [16]. Therefore, we grouped participants according to their self-reported sleep quality the night following the 20 mg dose of METH. Sleep quality was included in analyses as a 2-level between-subject variable based on a median split. Further, to study the specificity of METH’s effects on memory for stimuli of particular emotional arousal and valence, we also examined the size of its effect on responses to items from each valence category using individual one-way repeated-measures ANOVA. Because we expected dose-dependent increases in drug effects, linear main effects of drug dose are reported. Significant main effects of METH dose were followed by paired *t*-tests. Quadratic valence effects are reported because we were interested in investigating the extent to which the drug affected emotional pictures (both positive- and negative valence) more strongly than unemotional pictures (neutral valence). Alpha was set at *p =* .05 for all analyses.

**Emotional Processing and Confidence Data**. Participants’ subjective ratings of their positive, negative, and arousal responses to the pictures were analyzed for: 1) the studied pictures at encoding, 2) the studied pictures at retrieval, and 3) the distractor pictures at retrieval. METH did not significantly influence the ratings at either encoding or retrieval, and so these results are not discussed. Initial analyses of drug effects on confidence judgments revealed no systematic effects and are not described further. Memory data was also reanalyzed using individualized valence and arousal categories according to each individual participant’s valence and arousal ratings of the pictures. These analyses yielded analogous results to those obtained using normatively determined valence categories, and so only analyses obtained using pre-determined valence categories are reported.

**Subjective and physiologic measures**. Drug effects on subjective and physiologic state were analyzed using one-way repeated-measures ANOVA, with drug dose (0, 10, 20 mg) as the within-subject factor. As with memory task data, significant main effects of METH dose were followed by paired *t*-tests.

## Results

### Participant characteristics

Demographic characteristics and current substance use for the encoding and consolidation groups are summarized in Table 2. All participants were healthy, and most were in their early twenties, self-identified at ‘white’, and reported light to moderate use of alcohol and other drugs. The groups did not differ on any measured demographic characteristic (*p*s>.10).

**Table 2:** Participants’ demographics and recent drug use by group.

	Encoding (*n* = 29)			Consolidation (*n* = 31)		
	Mean	SD	Range	Mean	SD	Range
Age (years)	23.00	3.94	18–34	23.94	4.38	18–32
Body Mass Index	22.39	2.17	19–26	22.55	2.05	19–26
Education (years)	14.57	1.43	12–18	15.42	1.48	14–18
Caffeine (cups/day)	1.06	0.89	0–4	1.17	1.08	0–4
Nicotine (cigarettes/week) for occasional smokers*	2.42	2.06	1–5	2.65	5.93	1–14
Alcohol (drinks/week)	7.10	9.58	0–50	5.03	4.92	0–21
Cannabis (times/month)	1.58	2.43	0–10	0.90	1.80	0–8

*Note*. *occasional smokers *n* = 6 for encoding group and *n* = 5 for consolidation group.

Before analyzing METH effects on memory accuracy, the sample was grouped in terms of sleep quality based on a median split (Sleep: adequate [over 6 hours; n = 31], poor [6 hours or less; n = 29]). Adequate sleepers included *n* = 14 for the encoding group and *n* = 17 for the consolidation group, and poor sleepers included *n* = 15 for the encoding group and *n* = 14 for the consolidation group. Adequate and poor sleepers did not differ on any measured demographic characteristic (*p*s>.10).

### Memory task performance

In the placebo condition, both groups of participants remembered studied pictures with high accuracy (i.e., hit rates were high and false alarm rates were low), and there were no group differences in memory for pictures from any valence category (all *t*s<1.99; *p*s>.05). Participants’ subjective valence ratings of the pictures were appropriate to the designated valence categories based on normative data, and, as expected, both positive and negative pictures were rated as significantly more arousing than neutral pictures under placebo conditions (all *p*s<.001). Consistent with our previous data, both groups rated negative pictures as slightly, but significantly, more arousing than positive pictures in the placebo condition despite their being equated on normative arousal (*p*s≤.02).

### METH effects at encoding

**Fig 1:**
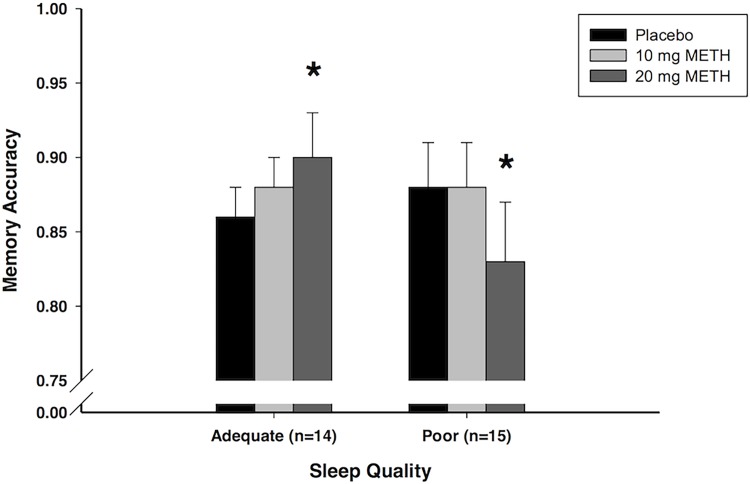
Meth effects on recognition. Effects of METH on mean (+/- SEM) recognition accuracy as a function of self-reported sleep quality in the encoding group. Adequate sleep group consists of participants who reported sleeping at least six hours the night after receiving the 20 mg dose (*n* = 14); poor sleep group consists of participants who reported sleeping less than six hours the night after receiving the 20 mg dose (*n* = 15). 1 = perfect discrimination; 0 = no discrimination (chance responding). *p<.05 compared to placebo

METH administered at encoding dose-dependently enhanced memory accuracy in those with adequate sleep, but impaired memory accuracy in those with poor sleep (Dose × Sleep interaction: *F*_1,27_ = 16.01, *p*<.001, *MSE* = 0.002, η_p_^2^ = .37; Fig. 1). Fig. 1 shows the METH-induced enhancement of memory accuracy in those with adequate sleep, and this was confirmed by a one-way repeated measures ANOVA (Dose: *F*_1,13_ = 7.76, *p* = .015, *MSE* = 0.002, η_p_^2^ = .37). Follow-up t-tests showed that METH (20 mg) significantly improved memory (10 mg vs. placebo: *t*_13_ = 0.88, *p* = 0.393; 20 mg vs. placebo: *t*_13_ = 2.79, *p* = 0.015). By contrast, METH impaired memory accuracy in the poor sleepers (Dose: *F*_1,14_ = 8.45, *p* = .011, *MSE* = 0.003, η_p_^2^ = .38). Follow-up t-tests showed that METH (20 mg) significantly impaired memory (10 mg vs. placebo: *t*_14_ = 0.06, *p* = 0.956; 20 mg vs. placebo: *t*_14_ = 2.91, *p* = 0.011).

**Fig 2:**
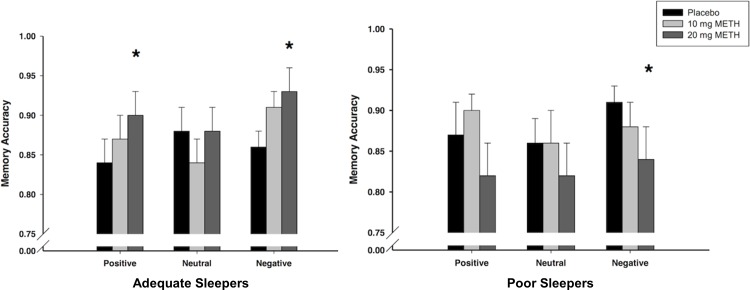
Meth effects on recognition by valence. Effects of METH on mean (+/- SEM) recognition accuracy for positive, neutral, and negative pictures among adequate sleepers (*n* = 14; left panel) and poor sleepers (*n* = 15; right panel) in the encoding group. 1 = perfect discrimination; 0 = no discrimination (chance responding). *p<.05 compared to placebo

While there was not a Dose x Valence interaction on memory, for comparison to our previous study in which AMP administered before encoding enhanced memory for emotional but not neutral pictures, we examined the effects of METH separately for positive, neutral, and negative pictures. Mean recognition accuracy scores for each valence in the adequate sleepers are presented in Fig. 2 (left panel). In line with our previous finding with AMP, METH (20 mg) improved memory for positive pictures (Dose: *F*_1,13_ = 7.81, *p* = .015, *MSE* = 0.003, η_p_^2^ = .38; follow-up t-tests: 10 mg vs. placebo: *t*_13_ = 0.96, *p* = 0.353; 20 mg vs. placebo: *t*_13_ = 2.80, *p* = 0.015). METH (20 mg) also improved memory for negative pictures (Dose: *F*_1,13_ = 6.55, *p* = .024, *MSE* = 0.005, η_p_^2^ = .34; t-tests: 10 mg vs. placebo: *t*_13_ = 1.66, *p* = 0.121; 20 mg vs. placebo: *t*_13_ = 2.56, *p* = 0.024). However, it did not alter memory for neutral pictures (*p* = .85). Mean recognition scores were also analyzed by valence in the poor sleepers, and these are presented in Fig. 2 (right panel). METH (20 mg) significantly impaired memory accuracy for negative pictures (Dose: *F*_1,14_ = 8.65, *p* = .011, *MSE* = 0.001, η_p_^2^ = .38; 10 mg vs. placebo: *t*_14_ = 1.26, *p* = 0.229; 20 mg vs. placebo: *t*_14_ = 2.94, *p* = 0.011) but not for positive or neutral figures (ps > 0.14).

### METH effects at consolidation

Table 3 presents memory accuracy following each dose in the consolidation group separately for adequate and poor sleepers. METH administered after picture viewing did not affect memory accuracy, and drug effects were unaffected by sleep quality, or by picture valence (*p*s > 0.10).

**Table 3:** Effects of METH on memory accuracy for each valence category and for all categories combined (Total) among adequate and poor sleepers in the consolidation group.

	Consolidation group							
	Adequate sleepers (*n* = 17)				Poor sleepers (*n* = 14)			
Dose	Positive	Neutral	Negative	Total	Positive	Neutral	Negative	Total
Placebo	.94 (.02)	.91 (.02)	.93 (.01)	.93 (.01)	.83 (.04)	.81 (.05)	.89 (.04)	.85 (.04)
10 mg METH	.95 (.02)	.91 (.02)	.94 (.02)	.93 (.02)	.81 (.05)	.81 (.06)	.85 (.06)	.82 (.06)
20 mg METH	.95 (.01)	.92 (.02)	.95 (.01)	.94 (.01)	.86 (.04)	.84 (.05)	.89 (.05)	.86 (.04)

*Note*. Data are mean recognition accuracy (SEM) for studied pictures. Participants were categorized as adequate sleepers if they self-reported 6 or more hours of sleep following the 20 mg dose, and they were categorized as poor sleepers if they self-reported less than 6 hours of sleep following the 20 mg dose.

### Subjective and physiological effects of METH

The timecourse of METH’s subjective, mood, and cardiovascular effects are summarized in Table 4 (encoding group) and Table 5 (consolidation group). Overall, consistent with its profile as a stimulant drug of abuse, METH dose-dependently increased subjective and physiological arousal and positive mood. As expected, initial effects were evident as early as 20 minutes after dosing, and most effects peaked at 40–60 minutes after administration. Subjective effects remained apparent for around 4–5 hours, whereas cardiovascular effects tended to persist longer (significant effects were still seen at the last timepoint in the encoding group—i.e., nearly 5.5 hours post-administration).

**Table 4:** Effects of METH on subjective and physiological measures in the encoding group (*n* = 29).

Measure	Placebo	10 mg	20 mg	Dose (*F*_1, 28_)
Feel—DEQ	1.37 (0.27)	1.58 (0.27)	2.75 (0.34)*	**15.02**
Like—DEQ	1.63 (0.33)	2.65 (0.44)	4.12 (0.41)*	**28.86**
Dislike—DEQ	1.28 (0.26)	1.2 (0.24)	1.23 (0.21)	0.03
High—DEQ	1.09 (0.27)	1.43 (0.28)	2.09 (0.31)*	**9.22**
Take Again—DEQ	2.82 (0.53)	4.18 (0.49)*	5.55 (0.46)*	**24.27**
Anxiety—POMS	-0.63 (0.47)	0.02 (0.44)	-0.24 (0.38)	.41
Elation—POMS	-1.94 (0.38)	-0.69 (0.48)*	0.31 (.48)*	**11.7**
Fatigue—POMS	0.79 (0.49)	-0.13 (0.37)	-0.44 (0.4)	4.16
Depression—POMS	-0.06 (0.13)	-0.29 (0.36)	-0.54 (0.5)	0.94
Confusion—POMS	0.7 (0.28)	0.01 (0.32)	0.28 (0.3)	1.36
Vigor—POMS	-3.37 (.66)	-1.28 (0.71)*	-1.04 (0.7)*	**4.97**
Arousal—POMS	-5.49 (1.26)	-1.14 (1.06)*	-1.12 (1.21)*	**5.46**
Positive Mood—POMS	-1.88 (0.41)	-0.4 (0.71)	0.85 (0.7)*	**10.83**
Systolic blood pressure (mmHg)	-3.32 (1.15)	4.84 (1.04)*	12.34 (1.99)*	**39.32**
Diastolic blood pressure (mmHg)	-1.25 (0.87)	4.81 (0.76)*	9.72 (1.04)*	**102.36**
Heart rate (bpm)	-4.56 (1.22)	2.92 (.98)*	5.54 (1.62)*	**25.61**

Data are change from pre-TX baseline means (SEM) averaged across time from +20 to +320 min post-capsule (with the exception of the DEQ [which was not administered pre-TX], and corresponding *F*-values from one-way repeated measures ANOVA examining linear main effects of drug dose. Bold values indicate a significant linear main effect of drug dose (*p*<.05).

*p<.05 compared to placebo.

**Table 5:** Effects of METH on subjective and physiological measures in the consolidation group (*n* = 31).

Measure	Placebo	10 mg	20 mg	Dose (*F*_1, 30_)
Feel—DEQ	-0.4 (0.26)	-0.01 (0.3)	0.8 (0.53)	3.62
Like—DEQ	-0.65 (0.28)	0.4 (0.37)*	1.42 (0.63)*	**8.98**
Dislike—DEQ	-0.61 (0.32)	-0.53 (0.38)	-0.11 (0.29)	0.86
High—DEQ	-0.12 (0.2)	0.05 (0.29)	0.66 (0.42)	2.15
Take Again—DEQ	-0.45 (0.26)	0.89 (0.35)*	1.9 (0.57)*	**16.26**
Anxiety—POMS	-0.14 (0.27)	-0.21 (0.85)	1.74 (0.57)*	**7.78**
Elation—POMS	-0.09 (0.32)	1.48 (0.5)*	2.77 (0.66)*	**19.37**
Fatigue—POMS	0.12 (0.39)	-1.21 (0.52)	-1.26 (0.58)	3.05
Depression—POMS	-0.16 (0.21)	-0.64 (0.22)	0.01 (0.28)	0.26
Confusion—POMS	-1.4 (0.53)	-1.38 (0.5)	-1.64 (0.74)	0.14
Vigor—POMS	-0.3 (0.56)	1.47 (0.57)*	4.52 (1.06)*	**20.21**
Arousal—POMS	0.84 (1.18)	3.85 (1.42)	9.15 (2.31)*	**15.21**
Positive Mood—POMS	0.07 (0.4)	2.12 (0.52)*	2.77 (0.8)*	**12.71**
Systolic blood pressure (mmHg)	-2.23 (0.96)	2 (1.13)*	5.25 (0.94)*	**29.68**
Diastolic blood pressure (mmHg)	-0.64 (0.89)	2.43 (0.82)*	5.03 (0.83)*	**18.78**
Heart rate (bpm)	-2.86 (1.29)	1.36 (0.91)*	1.94 (0.81)*	**13.42**

Data are change from pre-TX baseline means (SEM) averaged across time from +20 to +240 min post-capsule, and corresponding *F*-values from one-way repeated measures ANOVA examining linear main effects of drug dose. **Bold** values indicate a significant linear main effect of drug dose (*p*<.05).

*p<.05 compared to placebo.

## Discussion

This study investigated the effects of acute, moderate doses of METH on emotional episodic memory formation in healthy human volunteers, according to whether the drug was present during both encoding and consolidation, or only during consolidation. Participants received placebo or METH either before (encoding group) or immediately thereafter (consolidation group) viewing emotional and neutral images, and memory was tested 2 days later under drug-free conditions. Previously, we found that amphetamine (AMP) preferentially enhanced memory for emotional relative to neutral pictures that were initially studied while under the influence [22], but we did not distinguish between the two memory processes. Here, using a closely related drug, METH, we sought to replicate the improved memory reported previously, and to determine whether the drug had an effect specifically during early-stage consolidation. We found that METH can enhance declarative memory formation when administered before study, but that its effects can be masked by its tendency to disrupt sleep. That is, METH administered at encoding significantly enhanced memory in adequate sleepers, but impaired memory in poor sleepers. By contrast, when METH was present only during consolidation, the drug did not affect memory, regardless of sleep quality. METH induced its expected subjective and physiological effects, with initial effects detectable 20 min after dosing.

This study provides clear evidence that acute doses of METH can enhance long-term memory (i.e., for at least two days) of information encoded while under the influence in humans. We are aware of only one other study that examined the effects of acute METH on episodic memory in humans. In line with our results, in that study, healthy volunteers exhibited superior memory for noun words presented shortly after a single intramuscular injection of METH (0.3 mg/kg), relative to placebo, whereas memory was not altered when the words were presented immediately before dosing [34]. However, in that study memory retrieval testing took place while participants were still under the influence, making it impossible to differentiate the effects of METH on memory formation from its potential effects on retrieval. This is an important limitation, as we have recently shown that AMP increases memory retrieval errors at the same doses that enhance memory encoding [35]. Here we tested memory two days after drug exposure and learning, and again showed that METH improves memory when administered prior to encoding, but not when administered during consolidation only. The memory-enhancing effects of METH observed here are also consistent with reports that the closely-related drug AMP enhances episodic memory formation in healthy humans when administered immediately before encoding [7,22]. The observed effects are also consistent with research indicating that both METH and AMP similarly increase synaptic concentrations of dopamine and norepinephrine [36], as well as circulating levels of adrenocortical hormones [37,38], all of which can directly promote memory formation [39].

In the encoding group, METH preferentially enhanced memory for emotional relative to neutral pictures, an effect parallel to our previous study with AMP [22]. Emotional stimuli activate specialized systems in the brain for processing emotional information, including the amygdala, which likely prioritizes them for memory [40]. AMP is known to potentiate amygdalar responses to emotional stimuli in healthy adults [41], making it likely that the closely related drug METH would have a similar effect. Such preferential enhancement of memory for emotionally salient material could have important implications for the abuse potential of METH. Studies with laboratory animals have shown that stimulant drugs directly affect emotional memory systems that guide learning about stimuli that predict reward (e.g., food and sex); that is, they enhance learning and memory for stimuli associated with drug reward [1,3]. The current findings, along with our previous findings with AMP [22] suggest that stimulant drugs have similar effects on emotional memory systems in humans. Future studies are needed to determine the degree to which such effects on emotional memory might influence learning for drug-related stimuli specifically in humans, as this could help explain the maladaptive learning underlying increased drug cue reactivity observed in drug dependent individuals.

In the consolidation group, METH did not affect memory accuracy, regardless of sleep quality. These results are inconsistent with studies showing that alcohol, another commonly abused drug, enhances memory when it is administered during consolidation [42], as does cortisol [43], and other arousing procedures [44,45], but see also [46,47,48]. It is possible that methodological differences in study design influenced the differences in findings. For example, prior reports of the enhancing effect of alcohol on consolidation have often used incidental learning procedures, in which participants are not explicitly told that they would be performing a memory test [49,50]. Additionally, these studies often strictly limit participants’ exposure to any verbal stimuli during the consolidation period (e.g., restricting access to television, movies, and reading materials) to avoid any verbal interference with the consolidation process [50,51]. Despite these differences in methodologies, it is unclear why memory enhancement with METH was observed in the encoding group and not in the consolidation group. Previous reports show that METH can increase performance on tasks of vigilance, divided attention, and working memory [34,52,53], and it could be that these attentional effects specifically enhance the initial encoding process. However, these conclusions are limited by the fact that we examined the effects of pre- and post-encoding METH on memory in separate groups of participants. Future studies that contrast the effects of drugs administered pre- and post-encoding, in the same individuals, may reveal the relative contribution of these two different memory processes.

These findings indicate that a lack of restful sleep can dramatically impair the long-term explicit retention of memories. Several participants reported moderate-to-severe sleep disturbances after the METH sessions, particularly following the higher dose (20 mg). Because restful sleep is known to be critical for the consolidation of newly-formed memories into long-term stores [16,54], we grouped subjects based on a median split of hours of sleep following the 20 mg dose of METH and compared drug effects on memory accuracy in adequate and poor sleepers. In contrast to the METH-induced memory enhancement in adequate sleepers described above, METH administered at encoding dose-dependently impaired memory in poor sleepers. This is not surprising, given that sleep is well known to be critically involved in memory formation, and several studies have shown that disrupting sleep profoundly impairs long-term memory consolidation [16]. By contrast, METH administered only during consolidation did not impair memory in poor sleepers. Again, it is not clear why memory impairment was observed in poor sleepers in the encoding group and not in the consolidation group. However, when analyses were further restricted to participants in the consolidation group who reported the most severe sleep disturbances (i.e., less than four hours) memory impairment was evident (analyses not shown). Taken together, the present findings highlight the potential for METH to have either cognitively enhancing or impairing effects, and they serve as an important reminder that effects of drugs on sleep should be carefully considered when studying drug effects on memory.

The memory impairing effects of METH in sleep-disrupted individuals have important implications for those using METH for its cognitive enhancing properties. For instance, the popular use of stimulant drugs by college students to stay awake and study could have unintended opposite effects, resulting in impaired memory for information learned while under the influence. It is also important to note that the current study examined acute effects of METH, whereas the drug is often consumed chronically. Chronic METH use typically results in prolonged sleep disruption, and thus the cognitive impairment observed here in sleep-disrupted individuals likely contributes to impaired cognition following chronic METH use [54]. Future studies will be necessary to fully understand the associations between acute and chronic effects of METH on memory and sleep.

In summary, these results extend our previous finding that stimulants enhance encoding, especially for emotional events, but only if present at the time of study, where the drug can affect both encoding and consolidation. These findings provide new evidence that METH does not appear to facilitate consolidation if administered after encoding. The ability of amphetamines to strengthen memories for emotional events experienced while under the influence may strengthen memories for previous positive, or salient, experiences related to the drug-taking setting. The finding that sleep impaired memory adds to a growing literature on the importance of sleep on cognition in both research and clinical practice.

## Supporting Information

S1 Data Data for analyses presented here. (XLSX)
